# Supplementary material for: Effects of water stably-enriched with oxygen as a novel method of tissue oxygenation on mitochondrial function, and as adjuvant therapy for type 2 diabetes in a randomized placebo-controlled trial
Source: PLoS One. 2021 Jul 14;16(7):e0254619. doi: 10.1371/journal.pone.0254619 (PMC8279347; doi:10.1371/journal.pone.0254619)
Supplement: S1 File — (DOCX) [file pone.0254619.s002.docx]

**STUDY PROTOCOL**

**PROTOCOL TITLE:**

**E**LO Water In **D**iabetes Care For **E**nhancement Of Blood Sugar Co**n**trol (EDEN Study) – A DoubleBlinded Randomized Controlled Trial

**PROTOCOL NUMBER:**

EDEN/DM/2016

**PROTOCOL VERSION:**

2.0

**PROTOCOL DATE:**

23 August 2016

**TEST PRODUCTS:**

ELO Drinking Water

**PRINCIPAL INVESTIGATOR:**

Dr Khoo Joo Ching Joan

Chief & Senior Consultant

Department of Endocrinology

#### PARTICIPATING CENTRE

Changi General Hospital

2 Simei Street 3

Singapore 529889

**SPONSOR:**

ELO Water Pte Ltd (A Hyflux Group Company)

Hyflux Innovation Centre

80 Bendemeer Road

Singapore 339949

Study Protocol Number EDEN/DM/2016, Version 2.0, Dated 23 Aug 2016 Page 1 of 22

# STUDY PROTOCOL SIGNATURE PAGE

##### Protocol Title

ELO Water In Diabetes Care For Enhancement Of Blood Sugar Control (EDEN Study) – A Double-Blinded Randomized Controlled Trial

**Protocol Number**

EDEN/DM/2016

**Protocol Version / Date**

Version 2.0 dated 23 August 2016

##### Declaration of Sponsor

This protocol has been approved by ELO Water Pte Ltd (A Hyflux Group Company). The following signature documents this approval.

| ____________________________________ |  | _________________________ |
| --- | --- | --- |
| Dr Choy Mei Yee  Director of Clinical and Biomedical Research  ELO Water Pte Ltd |  | Date |

##### Declaration of Principal Investigator

I agree to conduct the described trial in compliance with all stipulations of the protocol, regulations and Singapore Guideline for Good Clinical Practice (SGGCP).

____________________________________ __________________________

Dr Khoo Joo Ching Joan Date

**TABLE OF CONTENTS**

[STUDY PROTOCOL SIGNATURE PAGE 2](#_Toc40766)

[1. BACKGROUND AND RATIONALE 5](#_Toc40767)

[1.1. RATIONALE AND JUSTIFICATION FOR THE STUDY 7](#_Toc40768)

[A. RATIONALE FOR THE STUDY PURPOSE 7](#_Toc40769)

[B. RATIONALE FOR DOSES SELECTED 7](#_Toc40770)

[C. RATIONALE FOR STUDY POPULATION 7](#_Toc40771)

[D. RATIONALE FOR STUDY DESIGN 8](#_Toc40772)

[2. HYPOTHESIS AND OBJECTIVES 9](#_Toc40773)

[2.1. HYPOTHESIS 9](#_Toc40774)

[2.2. PRIMARY OBJECTIVES 9](#_Toc40775)

[2.3. SECONDARY OBJECTIVES 10](#_Toc40776)

[2.4. POTENTIAL RISKS AND BENEFITS: 10](#_Toc40777)

[A. END POINTS – EFFICACY 10](#_Toc40778)

[B. END POINTS – SAFETY 10](#_Toc40779)

[3. STUDY POPULATION 10](#_Toc40780)

[3.1. LIST THE NUMBER OF SUBJECTS TO BE ENROLLED 10](#_Toc40781)

[3.2. CRITERIA FOR RECRUITMENT 10](#_Toc40782)

[3.3. INCLUSION CRITERIA 10](#_Toc40783)

[3.4. EXCLUSION CRITERIA 11](#_Toc40784)

[3.5. WITHDRAWAL CRITERIA 11](#_Toc40785)

[3.6. SUBJECT REPLACEMENT 11](#_Toc40786)

[4. TRIAL SCHEDULE 11](#_Toc40787)

[5. STUDY DESIGN 12](#_Toc40788)

[5.1. SUMMARY OF STUDY DESIGN 13](#_Toc40789)

[6. METHODS AND ASSESSMENTS 13](#_Toc40790)

[6.1. RANDOMISATION AND BLINDING 13](#_Toc40791)

[6.2. CONTRACEPTION AND PREGNANCY TESTING 14](#_Toc40792)

[6.3. STUDY VISITS AND PROCEDURES 14](#_Toc40793)

[7. TRIAL MATERIALS 15](#_Toc40794)

[7.1 TRIAL PRODUCT (S) 15](#_Toc40795)

[7.2 STORAGE AND PRODUCTS ACCOUNTABILITY 15](#_Toc40796)

[8. TREATMENT 15](#_Toc40797)

[8.1. RATIONALE FOR SELECTION OF DOSE 15](#_Toc40798)

[8.2. STUDY PRODUCT FORMULATIONS 15](#_Toc40799)

[8.3. STUDY PRODUCT ADMINISTRATION 15](#_Toc40800)

[8.4. SPECIFIC RESTRICTIONS / REQUIREMENTS 15](#_Toc40801)

[8.5. BLINDING 16](#_Toc40802)

[8.6. CONCOMITANT THERAPY 16](#_Toc40803)

[9. SAFETY MEASUREMENTS 16](#_Toc40804)

[9.1. SAFETY EVALUATION CRITERIA 16](#_Toc40805)

[9.2. COLLECTING, RECORDING AND REPORTING OF SERIOUS ADVERSE EVENTS (SAES) 16](#_Toc40806)

[9.3. SAFETY MONITORING PLAN 17](#_Toc40807)

[10. DATA ANALYSIS 17](#_Toc40808)

[10.1. DATA QUALITY ASSURANCE 17](#_Toc40809)

[10.2. DATA ENTRY AND STORAGE 17](#_Toc40810)

[11. SAMPLE SIZE AND STATISTICAL METHODS 17](#_Toc40811)

[11.1. DETERMINATION OF SAMPLE SIZE 17](#_Toc40812)

[11.2. STATISTICAL AND ANALYTICAL PLANS 17](#_Toc40813)

Study Protocol Number EDEN/DM/2016, Version 2.0, Dated 23 Aug 2016 Page 3 of 22

#### 12. ETHICAL CONSIDERATIONS ................................................................................................. 18

12.1. INFORMED CONSENT ................................................................................................................................. 18

12.2. IRB REVIEW ............................................................................................................................................... 18 12.3. CONFIDENTIALITY OF DATA AND PATIENT RECORDS ................................................................................ 18

#### 13. PUBLICATIONS ....................................................................................................................... 18 14. RETENTION OF TRIAL DOCUMENTS .................................................................................... 18 15. APPENDICES ........................................................................................................................... 20

Appendix 1 Water Delivery Process

**STUDY PROTOCOL**

# 1. BACKGROUND AND RATIONALE

#### General Introduction

*Study summary*

This study evaluates the efficacy of ELO water as an adjuvant modality in diabetes care for enhancement of glycemic control in patients with Type 2 diabetes mellitus.

##### *Diabetes – a major public health problem*

Global diabetes mellitus prevalence is at a staggering rate. The IDF reported that in 2015, a total of 415 million people suffer from diabetes and by 2040, this number is expected to rise to 642 million^1^. Out of the current 415 million people with diabetes, 320 million belong to the economically active age group of 20 -64 years^1^.

In 2015, IDF reported that Singapore has 12.8% of its population diagnosed with diabetes mellitus, a similar percentage as reported in the USA^1^. More than 90 percent are type 2 diabetes, which is generally considered to be contributed largely by lifestyle. Our Minister of Health has recently declared war on diabetes because the socioeconomic impact of diabetes on Singapore is monumental and is expected to escalate in the coming years.

##### *Diabetes management principles*

A prime care parameter that influences risks of diabetic complications is blood glucose control. Landmark studies such as the UK Prospective Diabetes Study (UKPDS)^2^ and Diabetes Control and Complications Trial(DCCT)^3^ have established that glycemic reduction holds the key to reducing risks of diabetic complications. As type 2 diabetes mellitus is a long term condition with a significant lifestyle component in its management, it stands to reason that finding effective lifestyle interventions to support drug therapy will help improve diabetes control. If there are lifestyle measures that are safe, convenient and form a part of normal living that can assist a diabetes patient in reducing blood glucose levels, these should be investigated as a potential aid in diabetes care.

##### *Oxygen, water and diabetes*

Oxygen is critically required by living cells. Water is equally important for cell survival. Hypoxia(lack of oxygen) and dehydration(lack of water) deprives cells of basic needs for oxygen and water and can lead to cell dysfunction and disease.

Oxygen improves the energy economics of the cell significantly. When cells are in a hypoxic environment, anaerobic processes are utilised by the cells to generate energy. The energy generating pathway under hypoxia produces only 2-4 ATP per molecule of glucose^4^. On the other hand, when oxygen is available to the cells, glucose is channelled into the mitochondria for oxidative phosphorylation and the electron transfer chain so that one molecule of glucose produces 32-36 ATP^4^.Oxygen vitally affects every cell type of the human body because every cell requires oxygen for energy generation to meet its metabolic needs.

A study done by researchers from Harvard Medical School, Ikazia Hospital, Rotterdam and Erasmus MC–University Medical Center, Rotterdam which was published in the Journal of Vascular Surgery demonstrated that diabetes patients (both type 1 and type 2) had 10 - 15% lower tissue oxygenation using transcutaneous oxygen (TcPO2) values obtained on the chest wall and limbs^5^. The study concluded that diabetes patients without signs of peripheral disease or neuropathy had significantly lower TcPO2 values compared with age- and sexmatched non diabetic patients^4^.*In vitro* studies on hypoxia has shown that that hypoxia creates a state of insulin resistance in fat cells through HIF (Hypoxia Inducible Factor) transcription factor expression^6^. Hypoxia could be mechanistic in creating insulin resistance in adipose tissue of diabetes or obese patients. A human trial by the University of Adelaide investigated the effect of increased tissue oxygenation on insulin sensitivity using the HOMA-IR method of euglycemic insulin clamp studies. This study found that when type 2 diabetes patients and overweight non diabetic patients were placed in a hyperbaric oxygen chamber, their insulin sensitivity improved^7^. Taken together, these studies suggest that there is a pathophysiological link between tissue oxygen levels and diabetes, possibly through mechanisms that involve insulin signalling and insulin resistance.

Cell processes occur in a ‘watery’ environment because 70 percent of cells is made of water. Within the living cells, water is used as a carrier of nutrients, electrolytes and oxygen for cell processes such as energy production, protein production and waste management.

Dehydration is a feature in poorly controlled diabetes and can aggravate hyperglycemia^8^ (high blood glucose) in diabetes patients. Chronic dehydration has been implicated as a factor in development of renal disorders including diabetic nephropathy^8^.A review published in the American Journal of Nephrology found that reducing vasopressin secretion, increasing water intake may have a beneficial effect on renal function in patients with all forms of chronic kidney disease (CKD) and may also benefit those at risk ofCKD^10^.The effects of good hydration on renal function of Type 2 diabetes patients should be explored as a simple lifestyle measure that could help ameliorate diabetic nephropathy.

*ELO water – what is it?*

ELO water is a pure drinking water that is enriched with molecular oxygen in a stable form. The ELO water looks and tastes like good quality drinking water without unusual taste or colour. It has passed the AVA standards for drinking water in terms of purity and mineral content and has been on sale commercially in Singapore since November 2015.

Oxygen naturally has low water solubility and has a tendency to immediately escape back into the air from solution. A special proprietary technology, the result of joint technology efforts by Hyflux Ltd and Kagun Europe Zrt Hungary, is able to produce ELO Water with a stable oxygen-enrichment. The oxygen content of ELO water is stable and is between 15 to 19 ppm, much higher than oxygen content in tap water (3-7 ppm)

A study by Ugolev has demonstrated that the mammalian gut lining is capable of oxygen absorption when his study demonstrated that oxygen saturated saline had 50-80% of the dissolved oxygen content absorbed when it was passed through 15 cm of rat gut^10^. Providing both hydration and oxygen, ELO water is an interesting type of water which potentially presents another platform to deliver oxygen and hydration which should be explored.

##### *Hypoxia Inducible Factor -1 alpha (HIF-1* α*)*

Systemic tissue hypoxia has been associated with increased plasma levels of HIF-

1α (Hypoxia Inducible Factor)^12^. In normoxic tissue conditions, HIF-1α is rapidly and continuously degraded following translation. Tissue hypoxia, however, induces a sustained increase in the expression of HIF-1α, measurable as plasma HIF-1α. This is mainly due to oxygen-dependent hydroxylation of HIF-1α amino acid proline residues in a reaction catalyzed by HIF prolyl-4-hydroxylases in the presence of its cofactors, oxygen and 2-oxoglutarate^13^. In human trials, it is technically more challenging to use PET scan and hypoxia radiotracers to establish change in tissue hypoxia levels, hence, we will be monitoring the plasma HIF-1α as an indication of systemic tissue hypoxia. This marker would indirectly help us monitor the effect of ELO water on the state of tissue oxygenation.

##### *Long chain acylcarnitines (LCAC)*

Diabetes patients and obese individuals have been observed to show higher plasma levels of long chain acyl-carnitines (LCAC).^14^Long chain acyl-carnitines are produced when there is incomplete mitochondrial fatty acid beta-oxidation (FAO) and this has been observed in diabetes patients. There are studies suggesting the role of LCAC in insulin signaling interference^15^, a potential mechanism for insulin resistance. Hypoxia itself has been shown to be associated with insulin resistance in adpocytes^5^. In a study involving dog muscle cells, hypoxia produced an accumulation of long chain acylcarnitines in the muscle cells^16^. It is possible that reversing tissue hypoxia in diabetes patients could enable the cellular mitochondria to completely breakdown long chainfatty acids through complete beta-oxidation and improve insulin resistance.

### 1.1. Rationale and justification for the Study

## a. Rationale for the Study Purpose

This study seeks to find a way to help diabetes patients obtain better glycemic control through drinking ELO water as part of the lifestyle intervention in diabetes care.

It is possible that orally taking in the oxygen carried in ELO water on a regular basis can supplement the conventional channel of oxygenation through the lungs and enhance oxygen availability to the cellular mitochondria. The hypoxia pathway to LCAC accumulation may be reduced using ELO water.^16^This could potentially improve fatty acid beta oxidation occurring in the mitochondria and reduce accumulation of long chain acylcarnitines (LCAC) in the plasma, which has been associated with interference with proper insulin signalling and increased insulin resistance^14,15^.

## b. Rationale for Doses Selected

It is suggested that 1.5 litres (1 bottle) of ELO water is to be consumed every day for a period of 24 weeks. Other forms of water must be used to supplement this in order to achieve the 2-3 litre total daily requirement of water in an adult.

24 weeks of daily ELO water consumption is a long enough period to ascertain changes in HbA1c.

## c. Rationale for Study Population

Rationale for inclusion criteria:

- The target population is adult Type 2 diabetes mellitus patients with HbA1c of between 8.0 – 11% in the 6 months prior to recruitment.

- The patients with this range of HbA1c have suboptimal glycemic control and allows the study to ascertain glycemic improvements.

Rational for exclusion criteria:

- Patients with comorbidities that require fluid restriction e.g. heart failure, renal failure are not suitable for studies that require oral fluid consumption of at least 1.5 litres per day.

- Pregnant patients are excluded from this study because at different stages of pregnancy, their insulin sensitivity changes.

## d. Rationale for Study Design

The study is a double-blinded, randomised and controlled trial (RCT) with 2 study arms, i.e. the ELO water arm versus the control arm with placebo drinking water. The control arm with placebo drinking water will define the biological effects attributable to ELO water with its higher oxygen content as opposed to effects due to general hydration. Blinding the patients to the type of water consumed will ensure that placebo effects are removed. The study investigators are also blinded to the type of water the patient is consuming to avoid study biases. The patients are asked to keep a water consumption diary as a way to improve monitoring of water drinking compliance.

**References:**

1. IDF Diabetes Atlas 2015
2. Paromita King, Ian Peacock, Richard Donnelly. The UK Prospective Diabetes Study (UKPDS): clinical and therapeuticimplications for type 2 diabetes.Br J ClinPharmacol 1999, 48, 643–648.
3. David M. Nathan, for the DCCT/EDICResearch Group.The Diabetes Control and Complications Trial/ Epidemiology of Diabetes Interventions and Complications Study at 30 Years: Overview. Diabetes Care 2014;37:9–16
4. Bodner, G. M. "The Tricarboxylic Acid (TCA), Citric Acid or Krebs Cycle." Journal of Chemical Education 63 (1986): 673-677.
5. Vincent E. de Meijer, Hans P.van’tSant, Sandra Spronk, FreekJ.Ksuters, Pieter T.den Hoed. Reference value of transcutaneous oxygen measurement in diabetic patients compared with nondiabetic patients.Journal of Vascular Surgery 2008;48:382-388
6. laire egazzetti, Pascal Peraldi, Thierry Gre m eaux, osanna NajemLendom,Issam en-Sahra,Mireille ormont, Fre de ric ost, annick Le Marchand-Brustel, Jean-Francois Tanti, and Sophie Giorgetti-Peraldi. Hypoxia Decreases Insulin Signaling Pathways in Adipocytes. Diabetes 2009; 58: 95103.
7. Wilkinson D, Chapman IM, Heilbronn LK. Hyperbaric oxygen therapy improves peripheral insulin sensitivity in humans. Diabet Med. 2012 Aug;29(8):986-9.
8. American Diabetes Association. Hyperglycemic Crises in DiabetesDiabetes Care 2004 Jan; 27(suppl 1): s94-s102.
9. N Bouby and S Fernandes. European Journal of Clinical Nutrition (2003) 57,

Suppl 2, S39–S46.

1. William F. Clark, Jessica M. Sontrop, Shi-Han Huang, Louise Moist, Nadine Bouby,LiseBankirHydration and Chronic Kidney Disease Progression: A

Critical Review of the Evidence.Am J Nephrol 2016;43:281–292

1. L.G. Ekkert, A.M. Ugolev.Influence of different modes of oxygenation of the small intestinal mucosa in vitro on glucose and maltose accumulation. Gen. Physiol. Biophys 1982; 2:141-157
2. William G. Kaelin, Jr.and Peter J. Ratcliffe. Oxygen Sensing by Metazoans:The Central Role of the HIF Hydroxylase PathwayMolecular Cell 30, May 23, 2008
3. Schuler B. et al.HIF-1 and the adaptation of man to high altitude.SchweizerischeZeitschriftfür «Sportmedizin und Sporttraumatologie» 53 (2), 82–87, 2005
4. Marieke G. Schooneman, Frédéric M. Vaz,Sander M. Houten,Maarten R. Soeters. Acylcarnitines. Reflecting or Inflicting Insulin Resistance?Diabetes 2013; 62:1-8.
5. Mihalik SJ, Goodpaster BH, Kelley DE, et al. Increased levels of plasmaacylcarnitines in obesity and type 2 diabetes and identification of a markerof glucolipotoxicity. Obesity (Silver Spring) 2010;18:1695–1700L.G.
6. Jane McHowat, Kathryn A Yamada, Jeffrey E Saffitz, and Peter B Corr. Subcellular distribution of endogenous longchain acylcarnitines during hypoxia in adultcanine myocytes.Cardiovascular Research 1993;27: 1237-1 243
7. Kim et al. Public Policy Committee of the American Association for the Study of Liver Disease. Serum Activity of Alanine Aminotransferase (ALT) as an Indicator of Health and Disease.HEPATOLOGY, Vol. 47, No. 4, 2008
8. Chetana N. Naresh, Andrew Hayen, Alexander Weening,Jonathan C. Craig, Steven J. Chadban. Day-to-Day Variability in Spot Urine Albumin-Creatinine Ratio.Am J Kidney Dis. 2013;62(6):1095-1101.

# 2. HYPOTHESIS AND OBJECTIVES

### 2.1. Hypothesis

Main Hypothesis

**Hypothesis 1**: ELO water as an adjuvant lifestyle intervention in Type 2 diabetes patients can improve glycemic control.

Secondary hypotheses

**Hypothesis 2:** ELO water, through its reversal of tissue hypoxia, is able to enhance mitochondrial beta oxidation of fatty acids, leading to reduced plasma levels of long chain acylcarnitines.

**Hypothesis 3:** ELO water, through a combined effect of hydration and oxygenation, is able to improve albuminuria in Type 2 diabetes patients better than hydration with regular water.

### 2.2. Primary Objectives

To evaluate oxygen-enriched ELO water as a viable aid for glycemic control when used as part of lifestyle management for Type 2 diabetes patients.

### 2.3. Secondary Objectives

1. To study the effect of drinking oxygen-enriched ELO water on mitochondrial fatty acid oxidation.
2. To study the effect of drinking oxygen-enriched ELO water on diabetic nephropathy.

### 2.4. Potential Risks and benefits:

## a. End Points – Efficacy

Primary end-point:

To achieve a reduction in HbA1c of at least 0.5% in 24 weeks

Secondary endpoints include the following:

1. Reduction in fasting blood glucose by at least 2 mmol/L
2. Reduction in fasting C14 or C16 or C18 long chain acyl carnitine by 25%
3. Reduction in any abnormally raised liver enzymes (GGT, ALT, AST) by more than 30%^17^
4. Reduction in calculated eGFR by 15 mL/min/1.73m^2^
5. Reduction in urine ACR in macroalbuminuric patients (urine ACR > 300 mg/g) of at least 48%^18^.

## b. End Points – Safety

Safety measures:

- Sponsor to deliver bottled water to patient’s home regularly, to avoid any danger to them if they carry heavy loads of water home.
- Hyflux is a public listed water company in Singapore with high quality standards for water products and ELO Water Pte Ltd is a Hyflux Group company. The ELO water has been approved by AVA Singapore for sale in Singapore since November 2015.

# 3. STUDY POPULATION

### 3.1. List the number of subjects to be enrolled.

Total 150 patients: 75 patients in ELO water arm and 75 patients in placebo water arm

### 3.2. Criteria for Recruitment

All patients must fulfil all of the Inclusion criteria (Section 3.3) and do not present with any of the Exclusion criteria (Section 3.4).

### 3.3. Inclusion Criteria

1. 21 years old to 70 years old inclusive
2. Type 2 diabetes mellitus patient
3. HbA1c ranging between 8.0% to 11% inclusive, within the last 6 months

### 3.4. Exclusion Criteria

1. Type 1 diabetes patients
2. Pregnant or lactating women
3. Comorbid conditions requiring fluid intake restriction
4. Patients who are unable to drink water due to inability to swallow or gastrointestinal conditions e.g. stroke or neurological disorders with inability to swallow fluids safely, bedridden patients with high risk of aspiration pneumonia, patients with oesophageal strictures, radical stomach resections or other conditions that makes drinking water unsafe

### 3.5. Withdrawal Criteria

Any patient will be withdrawn from the study if any of the criteria apply:

1. The patient withdraws consent
2. The patient is no longer able to participate , or the investigator, in discussion with the patient’s attending physician(s), decides to terminate participation on medical grounds

### 3.6. Subject Replacement

No subject replacement in this study.

# 4. TRIAL SCHEDULE

Table 1 in the following provides a flow chart presenting the study visit schedules and the study activities for the visits.

###### Table 1: Trial schedule

| **Week** | **0** | **6** (+/– 1 week) | **12**  (+/– 1 week) | **24**  (+/– 1 week) |
| --- | --- | --- | --- | --- |
| Consent taking | **X** |  |  |  |
| Inclusion &  Exclusion Criteria  Check | **X** |  |  |  |
| CGH clinic review^a^ | **X** | **X** | **X** | **X** |
| Vital Signs (BP) | **X** | **X** | **X** | **X** |
| Height and Weight | **X** | **X** | **X** | **X** |
| Blood and Urine tests^b^ | **X** | **X** | **X** | **X** |
| Completion of study |  |  |  | **X** |

*a :CGH review will be performed on Week 0, 6, 12, 24 and whenever there is any adverse events. Review will include review of water diary and to ask patient whether there has been any adverse effect, and / or changes in medications, diet and physical activities*

*b: Blood and Urine tests for HbA1c, Haemoglobin, Liver function test (ALT, AST, GGT), Serum creatinine and calculated eGFR, Fasting Lipid profile – Triglyceride, Total Cholesterol, LDL, HDL, Fasting plasma glucose, Plasma Hypoxia inducible factor HIF-1α, Long chain acyl carnitine (C16) and Urine ACR*

# 5. STUDY DESIGN

This is a randomized, controlled, patient and assessor-blind study, with 2 treatment arms in a single centre for 24 weeks. The 2 treatment arms are as shown on Table 2.

###### Table 2: Study arms

| **Treatment arm** | **ELO water** | **Placebo drinking water** |
| --- | --- | --- |
| Drinking 1.5 Litre water daily | ELO bottled water | Normal bottled drinking water |
| Diabetes treatment | Patient’s existing medications | Patient’s existing medications |

A total of 150 Type 2 diabetes mellitus patients who are eligible for the study will be enrolled into this 24 week study.

1. Each patient will be allocated a subject number for the study.
2. Once consented, patients who are recruited will be stratified according to their gender and will be randomised to the either of the 2 treatment arms (see table 2) in the ratio of 1:1.
3. Baseline blood tests (see table 3) will be done at a CGH clinic or CTRU.
4. Patients in both treatment arms will be issued water for drinking that is delivered to their home regularly (Bottled ELO water 1.5 litres or placebo bottled drinking water 1.5 litres) in non-identifying bottles that do not reveal to the patients as to which type of water they are consuming.
5. Study team member will remind patients regularly (via phone call or text message) to drink the study issued water, in order to archive the compliance.
6. Patients are instructed to consume 1.5 litres in divided servings per day. No metal containers or utensils to be in contact with the ELO water. The water cannot be heated or microwaved and should not be mixed with other beverages and consumed.
7. Patients are advised to drink other fluids or water in addition to the 1.5 litres of the bottled water to fulfil their daily water requirements of about 2-3 litres per day.
8. Patients are advised to strictly continue taking their prescribed existing medications and attend scheduled medical appointments throughout the study period.
9. No changes to be made in their diet, physical activity or medications during the study period.
10. Blood tests are performed at week 0, 6,12, and 24 (see table 3)
11. Patients are instructed to keep a water diary

###### Table 3: Laboratory Test at Week 0, 6, 12, 24 weeks

| **Test** | **Collection Tube** | **Volume** |
| --- | --- | --- |
| HbA1c | K^2^EDTA Tube | 2mls |
| Hb |  |  |
| Liver function test (ALT, AST, GGT) | SST Tube with clot activator | 5mls |
| Serum creatinine and calculated eGFR |  |  |
| Fasting Lipid profile – TG, TChol, LDL, HDL |  |  |
| Fasting plasma glucose |  |  |
| Plasma Hypoxia inducible factor HIF-1α | Lithium Heparinised Tube | 8-10mls |
| Long chained acyl carnitine | Lithium Heparinised Tube | 8-10mls |
| Urine ACR | Urine Collection Bottle | At least 10mls |

Special laboratory tests used in the study

1. Plasma HIF-1α

This will be done using an ELISA based test kit from ThermoFischer

Scientific.The Thermo Scientific™ HIF-1 Alpha ELISA Kit measures human hypoxia-inducible factor 1-alpha (HIF1A) protein in plasma using the quantitative sandwich ELISA method. HIF1A standards and samples are captured by a polyclonal HIF1A antibody on the precoated plate and detected using a biotinylated monoclonal HIF1A antibody reactive to epitopes other than the capture antibody. The biotinylated detection antibody is then bound to streptavidin-HRP, which catalyzes the conversion of TMB to a colored derivative. olour development is linear for the assay’s dynamic range and directly proportional to the amount of HIF1A present in the sample.

1. Plasma acyl carnitine assay

The plasma level of acylcarnitines of various carbon lengths will be measured using tandem mass spectrometry.

### 5.1. Summary of Study Design

- The study involves 150 Type 2 diabetic patients with HbA1c between 8% and 11% inclusive.
- 2 study arms – ELO water arm and Placebo drinking water arm  Reviews at CGH according to trial schedule in table 1
- Blood and urine tests are taken at week 0, 6, 12, and 24.

# 6. METHODS AND ASSESSMENTS

- Blood and urine tests
- Review of water diary
- The data will be stored in a study designated hard drive which will be retained for a period of 7 years and be erased after that.

### 6.1. Randomisation and Blinding

Randomization:

Stratified randomization will be performed for this study. Recruited patients will be stratified according to their gender and will be randomly assigned to ELO water arm or Placebo drinking water arm in a 1:1 ratio. A randomisation list will be generated by an independent biostatistician. Blinding of drinking water bottles will then be packaged according to the randomization list. Treatment allocation will be performed by the study nurse once all the recruitment criteria have been fulfilled and consent obtained.

Blinding:

ELO water Pte Ltd (A Hyflux Group Company) will blind the Hyflux factory bottled ELO water and Hyflux factory bottled Placebo drinking water by similar packing and label it as Water A or Water B, to unidentified drinking water bottles for both arms of the study. Water A or Water B for ELO water or Placebo drinking water will be pre-determined by the independent biostatistician.

Water bottles will be delivered to the patient’s home at regular intervals by an independent logistics team (who will not know what is Water A and Water B). A designated CGH staff member will co-ordinate with the logistics team for delivery of the correct type of bottled water to the patients. PI, attending investigators, study team members, and patients will not be able to identify the type of water the patient has been consuming.

### 6.2. Contraception and Pregnancy Testing

- Pregnant or lactating patients are excluded from the study
- Any patient who becomes pregnant during the study will be withdrawn from the study.
- This exclusion is because pregnant and lactating women have changes in their insulin sensitivity as a result of hormonal change

### 6.3. Study Visits and Procedures

###### *a. Screening Visits and Procedures (Week 0)*

Patients will be screened to determine eligibility for participation in the study. The following will be performed and documented at the Week 0 visit:

1. Obtain written informed consent
2. Review of Inclusion/Exclusion criteria
3. Baseline blood tests and urine tests
4. Review patient compliance to his/her existing medications

###### *b. Study Visits and Procedures*

Refer to Table 1 for the schedule of study review visits.

At each CGH review visit:

- Blood and Urine tests (table 3) will be performed as per scheduled
- Water diary record will be reviewed
- Patients are asked if they have any adverse reactions to the water assigned
- Body weight and height, blood pressure are taken

*c. Final Study Visit:*

- The scheduled final visit should occur after the patient has undergone 24 weeks of treatment or at the early withdrawal
- This visit includes a visit to CGH clinic/ CTRU.
- The week 24 blood tests (see table 3) will be performed at this visit.

# 7. TRIAL MATERIALS

1. Hyflux factory bottled ELO drinking water– 1.5 litre oxygen-enriched water
2. Hyflux factory bottled placebo drinking water – 1.5 litres

### 7.1 Trial Product (s)

Trial products include the following:

- ELO water for drinking is oxygen enriched water that comes in a 1.5 litre format
- Placebo drinking water in a 1.5 litre format
- Both bottles look similar and are non-identifiable

### 7.2 Storage and Products Accountability

- The ELO water and placebo drinking water should be stored at room temperature in a cool place away from direct sunlight.

- These study water will be stored at a third party commercial logistics vendor facility and delivered to the patients at regular intervals by the logistics vendor upon the request of the CGH study team.

- An outline of the water delivery process is attached in appendix 1

# 8. TREATMENT

### 8.1. Rationale for Selection of Dose

- ELO water for drinking – 1.5 litres a day in divided servings
- Placebo drinking water – 1.5 litres a day in divided servings
- Patients are advised to consume other forms of beverages or water in addition to the ‘1.5 litre study water’ to make up the recommended 2-3 litre per day for adults.
- The 1.5 litres of study water fulfils 50 – 75% of an adult person’s recommended daily intake of water and is a reasonable amount to ask the patient to drink

### 8.2. Study Product Formulations

- ELO water for drinking in 1.5 litre bottle.
- Placebo drinking water in 1.5 litre bottle.

### 8.3. Study Product Administration

Routes of administration of ELO regime:

1. Oral – drinking 1.5 litres of ELO or Placebo drinking water per day in divided servings in non-metallic vessels. No heating or boiling or microwaving of the ELO water.

### 8.4. Specific Restrictions / Requirements

- Patients advised to comply with their existing prescribed medications

- Patients are advised not to have changes in their diet, physical activity or medications during the 24 week study period

- Patients are advised not to add on any new traditional medications or health supplements during the 24 week study period

### 8.5. Blinding

Both patients and attending staff reviewing the patients during the study will be blinded to the treatment arm and the type of water the patient is consuming.

The Sponsor will manufacture the 2 types of bottled water (ELO and control) at their factory. The water will be in similar unidentifiable bottles with labels specifying the Water Code allocated by CGH. The Sponsor will be aware of which Water Code is for ELO water and which water is for control water.The Sponsor will not have any access to information of water allocation to patients (because the delivery is managed by a third party commercial logistics vendor.

The logistics vendor will deliver the water to the patient’s home when activated by CGH using Water Codes to identify which water to deliver. (see attached plan in appendix 1)

The independent biostatistician will un-blind the treatment allocated to the patients only after the completion of the study and for analysis purpose.

### 8.6. Concomitant therapy

All medications taken by the patient during the study period will be documented.

# 9. SAFETY MEASUREMENTS

### 9.1. Safety Evaluation Criteria

Clinical observation and laboratory results will be used to evaluate the wellbeing of the patient on a continual basis throughout the duration of the study.

In the event that the Investigator considers that the wellbeing of an individual patient is unacceptably at risk as a result of participation in the study then the individual patient will be withdrawn from the trial.

In the event that the withdrawal of an individual also has wider implications for the wellbeing of other participating patients, then the study will be suspended and an investigation conducted to determine whether the trial should be continued in the proposed or a modified form or terminated.

### 9.2. Collecting, Recording and Reporting of Serious Adverse Events (SAEs)

A serious adverse event (SAE) is any untoward medical occurrence that:

- Results in death.
- Is life-threatening (immediate risk of death)
- Requires inpatient hospitalization or prolongation of existing hospitalization.
- Results in persistent or significant disability/incapacity.
- Results in congenital anomaly/birth defect.
- Is a Medically important event.

All SAE will be collected, documented, assessed and reported by the PI to SHS CIRB according to its guideline, and to the Sponsor in the prescribed SAE form.

There are no known adverse effects of drinking the ELO water.

Complaints will be recorded in the case notes, and possible adverse events will be recorded in a separate section in the case records form (see attached).

PI and co-investigators will determine the severity, causality and expectedness of adverse events. If the investigator determines that the event may jeopardize the subject and/or may require intervention to prevent one of the other adverse event outcomes, the important medical event will be reported as serious. Causality will be defined as related/not related, and expectedness will be based on reported adverse events in the summary of product of characteristics.

### 9.3. Safety Monitoring Plan

There is no Data Safety Monitoring Board for this study. The PI and coinvestigators will perform data and safety monitoring. The PI will regularly monitor the study to verify the authenticity, accuracy and completeness of data, that the safety and rights of subjects are protected and that the study is conducted in accordance with the latest approved protocol, GCP and all applicable regulatory requirements.

# 10. DATA ANALYSIS

### 10.1. Data Quality Assurance

Data integrity will be monitored to ensure the accuracy, completeness and legibility of data collected. The frequency of data monitoring will be determined by the recruitment rate.

### 10.2. Data Entry and Storage

Case record forms will be stored in locked cabinets in the research office. Research data will be stored in the designated computer which is password protected. Only the delegated study staff will have access to the research data. The study participants' identification will be coded and their records are only available to the investigators.

# 11. SAMPLE SIZE AND STATISTICAL METHODS

### 11.1. Determination of Sample Size

We expect there is a medium effect size ( ohen’s d = 0.5) between ELO water arm and control water arm. The minimum number subjects to be recruited into each arm will be 65 subjects (total 130 subjects) for the study to have 80% power with significance level of 5%. Taking into account a potential dropout rate of 15%, we aim to recruit 75 patients in each arm (total 150 subjects).

### 11.2. Statistical and Analytical Plans

Categorical data will be presented as frequency (percentage). Continuous data will be presented as mean (SD), unless specified otherwise. The differences in characteristics will be examined using Chi-Square test or Fisher’s Exact test for categorical variables, and 2-sample t-test or Mann Whitney U test for continuous variables. For repeated measures variables, mixed models analysis will be used to account for drop-outs.

A two tailed, p-value of <0.05 was considered statistically significant. Statistical analysis will be performed with SPSS statistical software, version 19.0 (IBM Corp. Armonk, NY).

A statistical analysis for month 3 data is planned in order to study the short term outcomes.

This RCT will be analysed by both intention-to-treat (ITT) and per protocol (PP) approach. Patients’ non-compliance will be monitored via their water diary. The compliance rate will then be adjusted in the data analysis.

##### 12. ETHICAL CONSIDERATIONS

###### 12.1. Informed Consent

The investigator will explain the study in full to the patient according to the approved Informed consent form. The investigator will comply with the SGGCP Guidelines and to the ethical principles that have their origin in the Declaration of Helsinki.

A copy of the signed Informed consent form will be provided to the patient for his/her retention and reference.

###### 12.2. IRB review

The study will be submitted to the SingHealth Centralised Institutional Review Board (CRIB) for review and approval. The study will be initiated only after the I approval is obtained. The I ’s guidelines will be followed for the conduct of this study.

###### 12.3. Confidentiality of Data and Patient Records

Medical records of all subjects which are collected for study purposes will only be accessible to investigators. Subjects will be given study numbers and will not be identifiable to non-investigators. During the study, investigations will be done in individual rooms.

##### 13. PUBLICATIONS

The results could be presented in local and international conferences and submitted for publication to peer-reviewed journals, subjected to sponsor’s approval.

##### 14. RETENTION OF TRIAL DOCUMENTS

Records for all participants, including CRFs, all source documentation (containing evidence to study eligibility, history and physical findings, laboratory data, results of consultations, etc.) as well as IRB records and other regulatory documentation will be retained at Site in a secure locked cabinet in the CGH office.

After completion of the study the master records will be maintained by study site at the Sponsor’s expense at a secure location for a period of not less than 6 years from the completion or discontinuation of the study. Access to the information will require the approval of the PI or designated assignee.

**Appendix 1:** Water Delivery Process


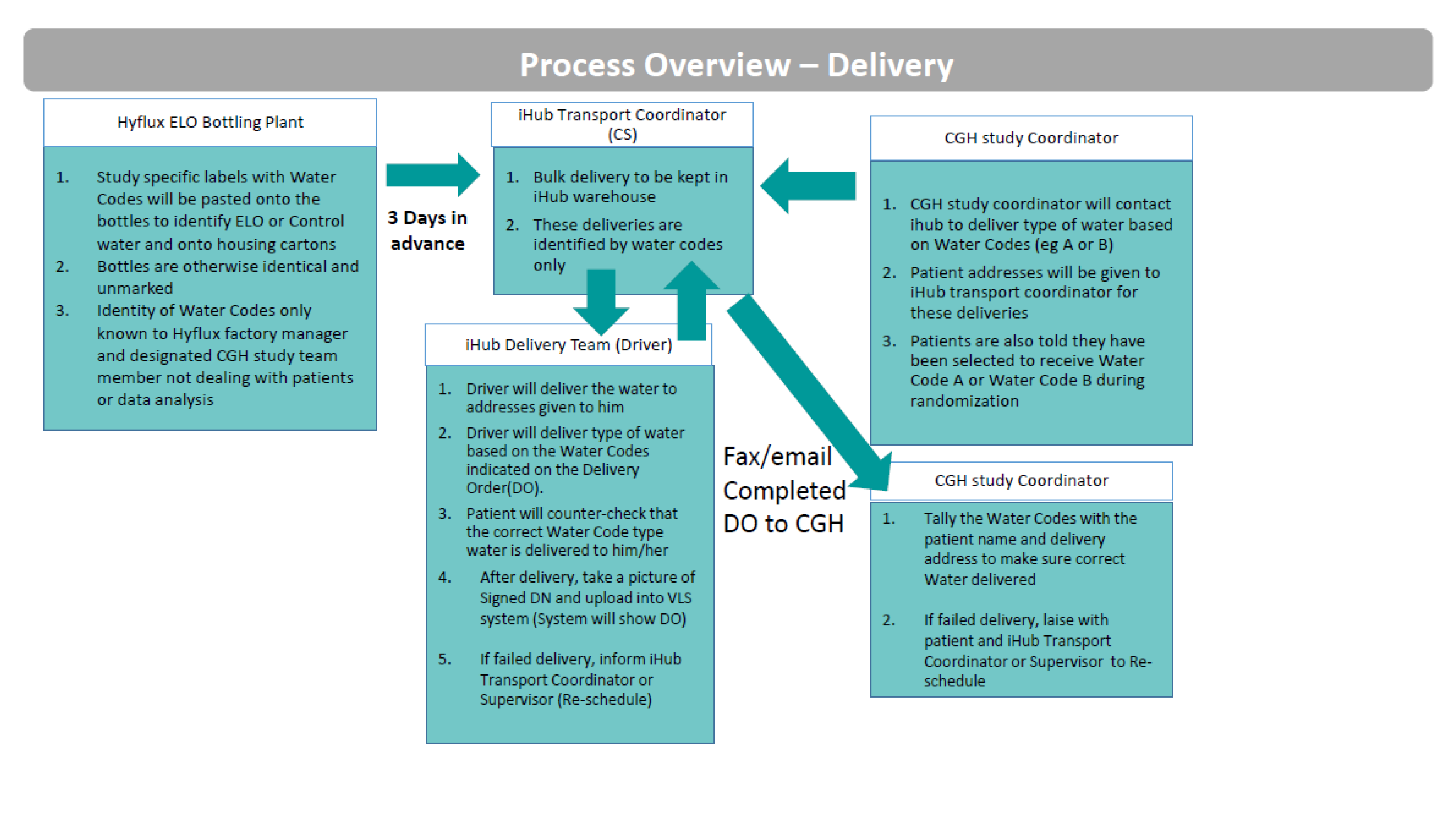
 Study Protocol Number EDEN/DM/2016, Version 2.0, Dated 23 Aug 2016 Page 22 of 22 F/CR/01-003.R3
