# Supplementary material for: Effects of water stably-enriched with oxygen as a novel method of tissue oxygenation on mitochondrial function, and as adjuvant therapy for type 2 diabetes in a randomized placebo-controlled trial
Source: PLoS One. 2021 Jul 14;16(7):e0254619. doi: 10.1371/journal.pone.0254619 (PMC8279347; doi:10.1371/journal.pone.0254619)
Supplement: Attachment — Submitted filename: PONE-D-20-22349R1 response to reviewers 090421.docx [file pone.0254619.s003.docx]

**PONE-D 20-22349: Effects of oxygen-enriched water on mitochondrial function, and as adjuvant therapy for type 2 diabetes in a randomized placebo-controlled trial**

**Response to Reviewers**

**Reviewer 1:**

1) This paper looks at a study in rats and a randomised controlled trial of ELO vs tap water in individuals with diabetes. At present the design characteristics of the randomised trial are not given - why was 150 participants chosen; how was randomisation performed etc.

**Response:** The reason for sample size of 150 participants was described in the section on Statistical Analysis i.e. taking the medium effect size (Cohen’s d = 0.5) between ELO arm and placebo, 64 subjects would be needed per group [ Cohen, J. (1988). Statistical power analysis for the behavioral sciences (2nd ed.). Hillside, NJ: Lawrence Erlbaum Associates] we recruited 75 subjects per arm (total 150) for the study to have 80% power with significance level of 5%, accounting for dropout rate 15%. We have moved this to the description of the sample size and randomization (page 9, lines 204-206).

The procedure for randomization has now been described on page 9, lines 206-210. Subjects were stratified according to their sex and randomized by an independent biostatistician using a computer program in a 1:1 ratio to two groups coded as A or B, to receive either ELO drinking water or bottled drinking water. The group assignations were concealed in sequentially-numbered opaque sealed envelopes. Investigators, subjects and study statisticians were all blinded to the code.

2) Please give exact p-values throughout and where t-tests etc have been performed give the difference and 95% CI. The dynamite plots currently give less information than a table with the actual data. Numbers are sufficiently small to give actual data points anyway - so the dynamite plots obfuscate the data.

**Response:** In the highlighted parts of the updated Results section (pages 12 and 13), absolute numbers and exact p-values have been provided for the oxygen consumption rate (OCR) for mitochondrial membrane proton leak in the in vitro stress tests, and in the new experiments on mitochondrial mass and membrane potential on page 14 (see below). We feel that the graphical representation of our results for 4 different cell culture environments (normal medium in normal-glucose; ELO medium in normal-glucose; normal medium in high-glucose; ELO medium in high-glucose) for each of 3 components of the mitochondrial stress test (Figure 4) is clearer than a table. Similarly, the newly added Figure 5 is a summary of changes in mitochondrial mass and membrane potential measurements for each of the 4 different cell culture environments.

3) The analyses in Table 1 are wrong. It is entirely incorrect to analyse within group - the between group differences and the p-values should be given and reported. Given the dropouts how many patients were analysed at each follow-up timepoint - was there any imputation of missing data?

**Response**: Between group-differences and p-values have been provided for Table 1 in the highlighted section. As described in the Statistical Analysis section (Page 10, lines 225-233), a general linear model with repeated measures and a one-sided significance level (P < 0.05) was used to evaluate within- and between-group differences from baseline to 6 and 12 weeks, with missing data imputed using a last observation carried forward-strategy.

4) Please remove the subgroup analysis unless a proper test for interaction demonstrates heterogeneity.

**Response**: A two-way interaction between the intervention and duration of diabetes was tested for using ANOVA, which was not statistically significant (p = 0.279). As such, the subgroup analysis has been removed.

**Reviewer 2**

1) The cell model chosen for the in vitro study is not appropriate. HepG2 cells are immortalized and metabolically are not at all similar to primary hepatocytes, and great care must be taken with these cells to make sure they are phenotypically similar to primary heps. There is no mention of these steps at all by the authors, and in any case, the rationale for using hepatocytes is weak, since these cells do not make sense to study in the context of T2 diabetes. Authors should choose consider using myocytes, adipocytes, or even pancreatic beta cells as a model, to determine if ELO has an impact on the metabolism of these cells. And this leads to the next comment,

**Response:** We agree that alternative cell lines such as myocytes, adipocytes, or pancreatic beta cells could have been used to study mitochondrial activity, and have included this as a limitation in the Discussion (page 18, lines 405-408).

However, hepatic insulin resistance is a major contributor to hyperglycaemia in type 2 diabetes (Reference 44, Iozzo P et al. Insulin-mediated hepatic glucose uptake is impaired in type 2 diabetes: evidence for a relationship with glycemic control. J Clin Endocrinol Metab 2003; 88:2055-60), and while it is true that HepG2 cells are immortalized and not primary hepatocytes, HepG2 cells have been used extensively in studies of mitochondrial function and in type 2 diabetes, NAFLD and other disorders of hepatic insulin resistance. This is because while Hep G cells are inherently different to primary hepatocytes, they are phenotypically stable and suitable for studies that are longer than several days. It is well known that there are major challenges in studying primary hepatocytes because they are very difficult to obtain, have limited in vitro proliferative capacities and quickly lose their hepatic phenotype and suffer poor viability in ex vivo environments (Reference 45, Ramboer E et al. Immortalized human hepatic cell lines for in vitro testing and research purposes. Methods Mol Biol 2015; 1250: 53–76).

Moreover, HepG2 cells are a better model than fresh human hepatocytes to measure mitochondrial function (Reference 46, Kamalian L et al. The utility of HepG2 cells to identify direct mitochondrial dysfunction in the absence of cell death. Toxicol In Vitro 2015; 29:732-40). We have included these reasons for using HepG2 cells in the Discussion (page 19, lines 409-413).

2) The increases seen in mitochondrial O2 concentration do not seem to be exclusive to ‘proton leak’, but rather all across the board, and this strongly suggests that ELO has simply increased mitochondrial content in these cells, which is not particularly surprising, but somewhat interesting. Authors need to provide evidence for changes in mitochondrial content in these cells.

**Response:** We agree that the increased mitochondrial O2 consumption was demonstrated in several measures, including basal respiration, which could reflect increased mitochondrial density, or function per mitochondrial unit. As mitochondrial turnover time is approximately 1-2 weeks in most cells, the treatment duration of 14 days in our study would have been sufficient to effect changes in mitochondrial content and/or function in these cells, hence we have performed further new experiments to quantify mitochondrial mass and membrane potential (a marker of hyperglycaemia-induced mitochondrial dysfunction, reference 33 Palmeira et al) as requested by the reviewer, now described on page 8 (lines 167-185), necessitating the addition of an author (R Xu) from the same laboratory.

There was no difference in mitochondrial mass at normal glucose concentration (5.5 mM) between cells incubated in ELO medium and those in control medium; in contrast, mitochondrial mass of the cells decreased when glucose concentration of control medium was raised to 25 mM, but was preserved in ELO medium at similarly high glucose concentration (Figure 5). Likewise, reduction in mitochondrial membrane potential of cells cultured in control medium (a marker of hyperglycaemia-induced mitochondrial dysfunction, reference 33 Palmeira et al; reference 34 Jiang et al) at glucose concentration of 25 mM was not seen in cells that were incubated with ELO medium in a similar high glucose environment.

The results of these experiments are reported on page 13, lines 289-300 and in the newly added Figure 5. We have discussed these findings in the Discussion (page 17 lines 356-370; page 18 lines 391-394) which suggest that ELO water appears to protect against hyperglycaemia-induced decrease in mitochondrial mass and mitochondrial dysfunction, which may be mechanisms by which glycaemic control of Type 2 diabetes patients was improved.

3) If the authors wish to make the argument that ELO alters redox environment or changes levels of oxidative stress in the body, or in the cells, then they need to provide evidence of this in their study. No markers or readouts of ROS or antioxidants were performed here at all.

**Response:** We thank the reviewer for highlighting this point, and have accordingly removed the references to protection against oxidative stress as a possible mechanism for improvement of mitochondrial function by ELO water from the Discussion. We have also incorporated the lack of measurement of oxidative stress markers in our studies as a limitation in the Discussion on page 18 line 402-3. However, this would require new experiments for which we do not have the funding.

4) Why did the authors not examine the effect of ELO more thoroughly in the rat model, specifically in a rat model of T2 diabetes? There are many options available, genetic- or diet-induced obese rat models, that are widely used. This would allow for a much more thorough interrogation of the effect of ELO.

**Response:** We agree that the findings of our in vitro studies would open the way for the effects of ELO water on oxidative stress and insulin resistance to be studied more thoroughly in animal models of type 2 diabetes, and thank the reviewer for this useful suggestion which we have added to the suggested future experiments in the concluding paragraph (page 19, lines 422-424). These animal experiments would require additional funding, which were not available after funding had been exhausted in the clinical trial on humans with type 2 diabetes.

5) The drop in HbA1c seen in the T2 diabetes patients with ELO water is somewhat interesting, but would be hard to argue that a drop from 8.9 to 8.6 is clinically meaningful.

**Response:** We have additionally analyzed the proportion of subjects with reduction in HbA1c of at least 0.5% (5.5 mmol/mol) at 6 weeks. This occurred in 39.1% (25/71) of the ELO water group, versus 18.9% (14/74) of the normal drinking water, which was statistically significant (p = 0.021). This has been added to the results section on page 15, lines 309-311, and discussed on page 17, lines 361-366.

Our study demonstrated a drop in HbA1c that is clinically significant and this quantum of HbA1c reduction is comparable to another study by Hirst JA et al where change in medication was used to induce a reduction in HbA1c. Ref 37, Hirst JA, et al, Changes in HbA1c Level over a 12-Week Follow-up in Patients with Type 2 Diabetes following a Medication Change. PLoS ONE 9(3): e92458),

6) It is disappointing that the authors did not measure fasting serum insulin levels in their pilot study, as this would have allowed them to determine if ELO has altered insulin sensitivity in the patients through the HOMA-IR index.

**Response:** Although HOMA-IR would have been useful information to obtain, we were concerned about the cross-reactivity of our insulin assay with exogenous insulin as about a third of our patients in both groups (similar proportions) were on basal or premix insulin. We have added the lack of information about insulin levels as a limitation in our Discussion on page 18, lines 403-405.
