# Supplementary material for: Effects of water stably-enriched with oxygen as a novel method of tissue oxygenation on mitochondrial function, and as adjuvant therapy for type 2 diabetes in a randomized placebo-controlled trial
Source: PLoS One. 2021 Jul 14;16(7):e0254619. doi: 10.1371/journal.pone.0254619 (PMC8279347; doi:10.1371/journal.pone.0254619)
Supplement: Attachment — Submitted filename: PONE-D-20-22349R2 response to reviewers 260521.docx [file pone.0254619.s004.docx]

**PONE-D 20-22349R2: Effects of water stably-enriched with oxygen as a novel method of tissue oxygenation on mitochondrial function, and as adjuvant therapy for type 2 diabetes in a randomized placebo-controlled trial**

**Response to Reviewer 1**

1) I understand the approach to build the trial based upon a standardised effect size - but such effect sizes are not necessarily the clinically relevant difference which is on the absolute scale. What absolute difference does d=0.5 correspond to, and why is this an appropriate choice?

**Response:** The medium effect size (Cohen’s d =0.5) was based on our hypothesis to detect a clinically significant absolute difference in achievement of HbA1c reduction of 0.5% between ELO and placebo arm with common SD of 1.0 using two-sample t-test (G*Power 3.1.9.7: Faul, F et al, G*Power 3: A flexible statistical power analysis program for the social, behavioral, and biomedical sciences. Behavior Research Methods 2007; 39: 175-191). We have clarified this in the revised manuscript (page 9 lines 204-205).

This reduction in HbA1c is clinically significant as it is the difference seen with change of dose/initiation of new medications over a period similar to that of our study (Ref 37, Hirst JA, et al, Changes in HbA1c Level over a 12-Week Follow-up in Patients with Type 2 Diabetes following a Medication Change. PLoS ONE 9(3): e92458).

2) It is well known (see the papers dating from the mid-1990s onwards) that envelope randomisation is prone to issues with allocation foreknowledge (indeed the classic case given by Altman in his textbook is an envelope randomisation). What steps were taken to ensure that envelopes were opened in sequence only after enrollment?

**Response:** An independent biostatistician who is not part of the study team was responsible for preparing the randomization numbers in individual sealed envelopes. Only one research co-ordinator was holding the envelopes. Each sealed envelope was opened in numerical order, in the presence of each subject only after he or she had signed the informed consent form, and the randomization number was then assigned accordingly. The dates of randomization were also recorded at the same time. With this process in place, each randomization number that was given out could be tracked using the date of informed consent, to ensure that it was given according to recruitment sequence. We have added this clarification into the revision (page 9 lines 211-215).

3) LOCF can be anti-conservative under certain circumstances - are results robust to method of imputing missing data?

Response: Handling missing data with the LOCF method was planned in the protocol *a priori*. The primary analysis of our data was performed using complete cases. We conducted sensitivity analysis using LOCF on the same dataset, which showed that the results and conclusion remained robust. This has now been added to the revision (page 10 lines 238-240).
